# Diseases of the Nervous System

**Published:** 1896-03-07

**Authors:** 


### Progress in Medicine.

### DISEASES OF THE NERVOUS SYSTEM.

**Epilepsy.**—The physiological pathology of epilepsy forms the subject of an important monograph by Marinesco and Sérieux.[1] They hold that the neurons of the cortex are of three kinds—the perceptive, the associative, and the centrifugal or discharging, and that there is not any direct route between the cortex and the muscles; every so-called voluntary, but really reflex, movement being made through the intermediation of the lower or spinal centres. The aura is simply a hallucination of cortical or reflex origin, according as the epilepsy is central or reflex in its nature. In the former the cortex plays the same part as does the epileptogenic zone in peripheral or reflex epilepsy. The loss of consciousness, which is an essential symptom of epilepsy, but which need not necessarily be complete, is explained variously by different authors, some attributing it to cerebral anæmia, some to congestion, and others still to functional interruption of the psychic elements (Herzen and Buccola), or to a disturbance of special centres (Bianchi). The authors consider it due to a phenomenon of arrest, bearing especially on the neurons of association. The stimulation of the perceptive centres produces the aura; that of both the perceptive and association elements produces automatism, pre- and post-epileptic delirium, &c.; that of the discharging neurons gives rise to the convulsions. The cry, which has been attributed to fear by Axenfeld, by Billod to spasm of the glottis and thorax, Marinesco and Sérieux attribute to excitation of the cortical laryngeal centre of Krause and Horsley. The fall is also one of the motor phenomenon, and is not due to the loss of consciousness. The pupillary immobility and dilatation is also a central phenomenon, as proved by the fact that if the sympathetic is divided on one side it fails on that side, while very marked on the other. The intense cerebral congestion is considered as a vaso-motor reflex from the sensory cortex. The view of Chaslin—that the original lesion in epilepsy is to be found in the neuroglia—has been investigated by Bleuler,[2] who examined twenty-six brains of epileptics on this point. He studied only the sub-pial proliferation of the neuroglia, which he found over the whole cortex, but the olivary region was not involved, as claimed by Chaslin. All the patients were more or less demented, and the changes corresponded more to the degree of dementia than to the epilepsy, and he could not determine the influence of the attacks in producing the lesion. Then eurolgia cells of the cortex were not increased in number, but were often pigmented.

Gilles de la Tourette[3] remarks that, anatomically, the epilepsy of young subjects appeared to be *sina materia*, but careful investigation of the various forms both macroscopical and microscopical, tuberous or neuroglic, of cerebral sclerosis has shown that the older views must be revised. On the other hand, the result of autopsies in certain cases of epilepsy in adults, which were assumed to be invariably due to osseous, meningeal, or other lesions, is frequently as negative as that of autopsies in patients suffering from the most characteristically idiopathic epilepsy. Heredity was formerly regarded as the etiological factor of idiopathic epilepsy, as a transformation of a neuropathic state of the parents into this condition, the direct transmission of the disease to the offspring, which is of frequent occurrence in hysteria, being but rarely met with in epilepsy. Nowadays heredity is looked upon as a predisposing cause, brought into play by various agents, among which Tourette calls attention particularly to traumatism of the skull at birth. If the resulting meningeal hæmorrhage does not prove fatal the clot is absorbed, but not infrequently this absorption determines on the subjacent cortical layers an irritative sclerotic process the germ of later epilepsy, the first manifestation of which soon supervenes in the form of infantile convulsions. Much light is thrown on the etiology of epilepsy by inquiry into the morbid history of the patient during childhood with reference to infectious diseases, such as scarlet fever in the first place, then measles, small-pox, enteritis, or broncho-pneumonia. The cerebral manifestations of these infections, which, when they reach their maximum degree of intensity, determine infantile spasmodic hemiplegia in consequence of extensive cortical sclerosis, are confined, when less marked, to neuroglic sclerosis, giving rise to continuous irritation of the motor cells clinically manifested later on by a fully developed epileptic seizure. The influence of heredity, as a factor in epilepsy, has thus been reduced to its true limits by more accurate investigations. The question naturally presents itself why a cerebral disease existing from the earliest years remains latent, in the majority of cases, until later childhood? The answer to this question, continues Tourette, is that the calm is more apparent than real, that the period of two, four, or six years and more which elapse between the original cause and the first attack, is not free from premonitory manifestations of the disease which should be carefully inquired into. On analysing the mental condition of these future epileptics, they will almost always be found to be of an irritable and violent temper, running angrily away, clenching their fists and grinding their teeth on the slightest provocation. Sometimes they have a fainting fit, the face becomes flushed, and the eyes are fixed in a convergent squint; this is the inauguration of a convulsive crisis which soon makes its appearance in an unmistakable form. Nothing like this occurs in subjects destined to become hysterical later on, these in contra-distinction from the former, as the author has shown, are apt to develop somnambulism. Later on momentary loss of consciousness supervenes; the patient retaining an upright position, interrupts the act on which he is engaged, or continues it automatically, becoming pale, motionless, and with a fixed look; but he promptly regains consciousness. These and other phenomena of epilepsia minor become associated later on with E. gravior. This gradation of symptoms is almost invariably observed in the epilepsy of young people, but is very rarely met with in adults in the form of the disease which makes its appearance later in life. In adults the affection is usually ushered in in a more dramatic manner by a severe attack familiar to everyone.

Tourette mentions the features distinguishing epilepsy from hysteria. The initial cry of epileptic attacks is not repeated, epilepsy differing widely in this respect from hysteria, in an attack of which the patient keeps on screaming, and these screams are characteristic. The stertor and snoring of epilepsy do not cease at once, and the patient, after the return of consciousness, does not completely recover, and remains dull and sleepy, with lassitude and severe headache. It takes from twenty-four to thirty-six hours for the patient to recover from the shock if the attack has been at all severe. Hysterical patients, on the contrary, after having been for hours the prey of the most extraordinary convulsions, resume their usual occupations, once the crisis is over, as if nothing had happened. A typical epileptic fit differs from an hysterical attack principally in three points—sudden fall, not preceded by aura, with complete loss of consciousness; biting of the tongue; involuntary emission of urine. In hysterical attacks a sudden fall is extremely rare. As a rule, the paroxysm is preceded by a cephalic aura; throbbing at the temples, tinnitus aurium, and transient hallucinations, which are more rarely absent than the well-known phenomenon described as globus hystericus. Biting of the tongue and involuntary emission of urine are still more exceptional phenomena in pure hysteria. But hysteria and epilepsy may co-exist in the same patient. In doubtful cases the urine passed during the twenty-four hours following the seizure should be carefully analysed, for, as Lépine and Mairet have shown, all the solid elements of the urine increase for twenty-four hours after an epileptic attack, whereas, in hysteria, as Cathebineau and the author have pointed out, the proportion of solids is sub-normal, the quantity of urine undergoing no alteration in either case. If we take the urea alone, the normal excretion of which is 25 grammes, it will be found after an epileptic seizure to be from 35 to 40 grammes, and after an attack of hysteria from 12 to 15 grammes in the twenty-four hours. Moreover, in epilepsy the ratio of earthy to alkaline phosphates, which is normally 1:3, remains unchanged, whereas in hysteria it is as 1:2 or 1:1, *i.e.*, in hysterical attacks there is what Tourette has called inversion of the proportion of phosphates added to the diminution in the quantity of solid residue—urea, chlorides, sulphates, &c. Ch. Féré[4] has described an interesting case, which illustrates the fact demonstrated by Lucian and Tamburini, that those cerebral centres are most excitable which correspond to the muscles most often brought into action. A woman, aged 32, belonged to a family of artisans free from neuropathic antecedents. She was well until the fourteenth year, after which, at several successive menstrual periods, she suffered from one or two convulsive seizures, in which she suddenly fell backwards with stiff arms, clenched fists, and turned-in thumbs. Attacks appeared at almost every period till her eighteenth year, when they disappeared without assignable cause till the twenty-eighth year. After the birth of a second child she began to work in a burnisher's shop, and continued working there for four years. A year later, at age twenty-eight, she was, during a menstrual period, attacked in the morning while dressing, and her husband noticed that before she fell she went through all the movements of her avocation with her right hand. Subsequent attacks always began with sudden loss of consciousness, and burnishing movements of the right hand. Both her husband and her sister insisted particularly, and independently of each other, upon the different effect upon the hand at the beginning and during the generalisation of the attacks. They dwelt upon the fact that at first the little finger was more flexed than the others, and the thumb was outside, and this was exactly the way the tool was held at work. From the moment the patient fell the attitude of the hand changed, and were those ordinarily seen in epilepsy. The patient possessed no hysterical stigmata.

Naunyn[5] records cases of three men, all above the age of sixty-three, with a history of epileptic attacks, in whom it was possible to bring on attacks precisely similar by compression of the carotids. He assumed, therefore, that the spontaneous attacks were due also to cerebral anæmia, which, on account of the weak heart and arterio-sclerosis existing in the patients, was not far to seek. Among other signs of anæmia of the brain, in the spontaneous attacks as far as they were observed, there was always a slow pulse. In one case an attack came on when the patient rose suddenly; in another case after a warm bath. Naunyn made experiments with carotid compression in persons who had never had epileptiform attacks, and who had no signs of brain disease or disturbance of the circulation. In persons not more than thirty years old compression of both carotids caused at most temporary dizziness. In two men over fifty, with arterio-sclerosis and slight disturbance of the circulation, but without brain symptoms, compression was followed in a few seconds by loss of consciousness, slow pulse, and mild general convulsions. So far as made, the observations seem to prove that senile epilepsy depends on circulatory disturbances, and to disease of the heart or vessels.

**Treatment.**—The Flechzig treatment of epilepsy by opium (given in half-grain doses and increased by one-half grain per day until 15 grains have been reached, when the opium is discontinued and the mixed bromides, 30 grains four times a day, are substituted) has been investigated by Isabel Davenport, M.D.,[6] in eleven patients, with similar results in all—cessation of the attack, but no permanent benefit. After a careful and thorough trial, extending over a period of more than a year, she is forced to conclude that: (1) Flechzig's method of treatment for epilepsy does not result in recovery; (2) that it is of benefit, in that it gives many of these unfortunates a gratifying respite from the attacks, and thus adds to their comfort; (3) that it is soothing and quieting to the irritable patients and exhilarating to those suffering from depression, thus relieving distressing symptoms in both cases; (4) that through the cessation of the seizures and other annoying symptoms the patient is enabled to enjoy something of life in general and to recuperate physically, and for these reasons the authoress believes it is desirable to repeat it at intervals of two or three months, if thereby we can obtain the results mentioned above. De la Tourette,[7] whose large experience renders his opinions of especial value, is a firm believer in the treatment employed by Professor Charcot. He regards it as an axiom that the only really efficacious remedy in epilepsy is bromide of potassium. Charcot was in the habit of employing simultaneously, in equal doses, the bromides of potassium, sodium, and ammonium, a combination very highly recommended by Brown-Séquard, but he never eliminated from the mixture bromide of potassium, as there is a tendency to do nowadays, bromide of strontium or sodium being substituted for it, to the exclusion of the other bromides. It has been alleged that bromide of potassium is the most toxic of all the bromides, but "Is it not a therapeutical intoxication we are striving to obtain?" asks Tourette. He states that one does not succeed in stopping the seizure until you are on the verge of toxic symptoms. Many failures are attributable precisely to this fear of intoxication. Tourette by choice gives ten to fourteen grammes daily, but never orders very small or slowly progressive doses, nor does he ever under any circumstances suspend the treatment under pretext of allowing the organism to rest for a time, or to accustom it to the bromide. The remedy must be administered as long as the treatment lasts, without a single day's intermission. This is Charcot's injunction, and the precept, says Tourette, is a valuable one. On account of possible susceptibility to the toxic effects it is necessary to proceed with caution, and if the patient has not already taken bromides it is well to administer progressively increasing or decreasing doses, giving five grammes daily during the first week, six grammes daily during the second, and seven grammes during the third. When a dose of seven grammes is reached the effect should be watched. One must not be over anxious at this time in obtaining an improvement in the attacks, for this always takes some time to accomplish, but pay particular attention to any ill-effects. If towards the end of the third week, when seven grammes are being taken, the patient's mind should be somewhat clouded, with a tendency to drowsiness, though he is still able to attend to his duties, the efficacious dose has been reached. Ultimately with the establishment of tolerance eight grammes may be given. The hour for administering the bromide should be regulated by the time of the seizures, when, as is the rule, they occur periodically. If the attacks are nocturnal or matutinal, and the patient is taking six grammes daily, four or five grammes should be given in the evening on retiring, and the rest in the morning. If the attacks occur at noon four grammes are given at nine in the morning, and the rest on retiring for the night. In other words, two-thirds of the dose should be given two or three hours before the probable appearance of the seizure. The drug should always be given in aqueous solution, one gramme to each teaspoonful of water, and taken in a glass of water or milk, and sweetened with sugar. The stomach is much better able to bear a diluted solution. If the drug produces gastro-intestinal disturbances, Tourette gives salol, ten centigrammes with each gramme of the three bromides. Bromide acne should be controlled by the prolonged application of the boracic spray morning and evening, and a soap bath ordered every ten or twelve days. The diet should be light and tonic, but there are no further special rules. The patient should go to bed at ten, and rise at six or seven a m. The evening meal should be very light if the attacks are nocturnal.

If one finds that the disease is retrogressing, it shows unquestionably that the dose of the remedy may be diminished until the treatment is ultimately discontinued altogether. Unfortunately, however, the paroxysmal symptoms of epilepsia, gravior or mitior, do not always yield completely to bromide, but attacks of momentary confusion supervene from time to time, and then the bromide must be continued; and in cases of this kind an effort should be made to ascertain the minimum dose, which cannot be reduced without exposing the patient to the risk of attacks.

When, as in a few cases, bromides are without much effect, Tourette gives borate of sodium, but without ever abandoning bromide of potassium altogether. The borate is given in doses of three to one gramme.

[1] Gaz. Hebdom. Nov. 23, 1895; Amer. Jour. of Insan. Jan., 1896. [2] Munich Med. Woch., Aug. 13, 1895; Amer. Jour. of Insan.; loc. cit. [3] Med. Week, Oct. 26, 1895. [4] Extrait des Comptes Rendues des Séances de la Société de Biologiè; edit. Med. Bul'etin, Dec.. 1895. [5] Zeitschr. f. Klin. Med. Bd. xxviii., p. 217; Amer. Jour. of Med. Sc., Nov., 1895. [6] Amer. Journ. of Insan. Oct., 1895; [7] Loc. cit.

